# Family phenotypic profile in hereditary hemorrhagic telangiectasia: genotype-phenotype correlation in a pediatric Italian population

**DOI:** 10.1186/s13052-025-02087-4

**Published:** 2025-09-24

**Authors:** Valentina Giorgio, Chiara Di Foggia, Giovanna Quatrale, Gaia Margiotta, Giuseppe Stella, Francesco Proli, Chiara Leoni, Roberta Onesimo, Giulio Cesare Passali, Andrea Contegiacomo, Giuseppe Zampino, Emanuela Lucci Cordisco, Elena Sonnini, Antonio Gasbarrini, Eleonora Gaetani

**Affiliations:** 1 Dipartimento di Scienze della salute della donna, del bambino e di sanità pubblica, UOC di Pediatria, Fondazione Policlinico Universitario A. Gemelli IRCCS, Università Cattolica del Sacro Cuore, Roma, Italia; 2 Dipartimento Materno Infantile e Scienze Urologiche, UOC di Pediatria, La Sapienza Università di Roma, Roma, Italia; 3 Dipartimento NESMOS, Facoltà di Medicina e Psicologia, UOC di Pediatria, Università Sapienza di Roma, Azienda Ospedaliero Universitaria Sant’Andrea, Roma, Italia; 4 Dipartimento di Neuroscienze, Organi di Senso e Torace, UOC di Otorinolaringoiatria, Fondazione Policlinico Universitario A. Gemelli IRCCS, Università Cattolica del Sacro Cuore, Roma, Italia; 5 UOC di Radiologia d’Urgenza e Interventistica, Dipartimento di Diagnostica per Immagini, Radioterapia Oncologica ed Ematologia, Fondazione Policlinico Universitario A. Gemelli IRCCS, Università Cattolica del Sacro Cuore, Roma, Italia; 6 UOC di Genetica medica, Fondazione Policlinico Universitario A. Gemelli IRCCS, Roma, 00168 Italia; 7 Department of Medical and Surgical Sciences, UOC CEMAD Centro Malattie dell’Apparato Digerente, UOC Medicina Interna e Gastroenterologia, Fondazione Policlinico Universitario Gemelli IRCCS, Roma, Italia; 8 Ospedale Cristo Re, UOC di Medicina Interna, Università Cattolica del Sacro Cuore, Roma, Italia

**Keywords:** Hereditary hemorrhagic telangiectasia, Pediatrics, Genotype-phenotype correlation, Anemia, *ACVRL1*, *ENG*, HHT

## Abstract

**Background:**

Hereditary Hemorrhagic Telangiectasia (HHT) is a rare, hereditary, autosomal dominant vascular disease, characterized by visceral arteriovenous malformations (AVMs), mucocutaneous telangiectasias and epistaxis. While symptomatic medical and surgical therapies are used in the management of pediatric patients, data comparing phenotypic presentation between genetically related individuals (e.g., parent-child pairs) remain limited.

**Aims and Methods:**

We conducted a single-center retrospective study involving pediatric patients with genetically confirmed HHT and their affected parents at Fondazione Policlinico Universitario A. Gemelli IRCCS in Rome, Italy. The aim of our study was to assess genotype-phenotype correlation and intrafamilial HHT-phenotypic variability. Clinical, laboratory, imaging, and therapeutic data were collected between May 2022 and May 2023. Variables including presence of AVMs, epistaxis, telangiectasias, anemia, and need for interventions were compared between children and their parents.

**Results:**

The study included 11 children (mean age 11.8 years) and 9 adults (mean age 47 years), all exhibiting *ENG* or *ACVRL1* variants. While epistaxis was common in both cohorts (91% in children vs. 100% in adults), mucocutaneous telangiectasias and AVMs were more prevalent in adults. AVMs were more frequently detected in adults, especially among *ENG* carriers. Notably, phenotype concordance between parent-child pairs with the same mutation was observed in only 3 up to 9 families (33.3%), with substantial intrafamilial variability in AVM distribution and disease severity.

**Conclusions:**

Our findings confirm the variable expressivity of HHT, even among first-degree relatives sharing the same pathogenic variant. No consistent overlap was observed between parent and child phenotypes, reinforcing the need for individualized pediatric screening and follow-up. Early genetic testing remains essential to prevent complications and guide appropriate management.

## Introduction

Hereditary Hemorrhagic Telangiectasia (HHT - OMIM number #187300) or Rendu-Osler disease is a rare, hereditary, autosomal dominant vascular disease (overall prevalence between 1/5000 and 1/8000 individuals). It is characterized by abnormal angiogenesis leading to the development of visceral arteriovenous malformations (AVM) and mucocutaneous telangiectasias, with epistaxis being the most frequent manifestation [1–5].

The genetic mutations responsible for HHT are primarily located in three genes:

- ENG (Endoglin): Located on chromosome 9, mutations in ENG are associated with HHT type (1) Endoglin is a co-receptor for transforming growth factor-beta (TGF-β), crucial for vascular development and integrity;
- ACVRL1 (Activin A receptor-like kinase 1): Found on chromosome 12, mutations in this gene lead to HHT type (2) ACVRL1 is also involved in TGF-β signaling pathways and plays a significant role in endothelial cell function;
- SMAD4 (SMAD family member 4): This gene is associated with a rare form of HHT that can present with juvenile polyposis syndrome. Mutations in SMAD4 disrupt the TGF-β signaling pathway, affecting vascular development.

Symptomatic medical and surgical therapies are used in the management of pediatric HHT patients, as outlined in the Second International Guidelines [6].

This is based on available literature [7–10] that presents the limits of a rare disease. According to expert panel, HHT pediatric patients should be tested for iron deficiency and anemia just in case of recurrent bleeding and/or symptoms of anemia, with hemoglobin targets individualized on patients (depending on symptoms, severity of HHT-related bleeding, previous iron supplementation, comorbidity). As regard to pulmonary AVMs, the expert panel recommends screening in children at the time of symptoms and then every 5 years in asymptomatic patients; it (screening) may be performed with chest radiography and pulse oximetry or trans-thoracic contrast echocardiography, while lung CT remains a confirmatory test, if screening tests are positive. Large pulmonary AVMs (at least 3 mm in diameter) or AVMs causing hypoxemia are suitable for embolotherapy to avoid complications. Finally, brain AVM should be screened at the time of presentation with MRI, representing the first-line screening to detect the presence of malformations and to determine the risk of hemorrhage. At the moment, there is no agreement if routinely screening for brain AVMs in asymptomatic children is recommended. Abdominal ultrasound for liver AVMs screening is performed according to adult recommendations [6].

Finally, considering the higher risk of polyps and colorectal cancer in SMAD4-HHT, screening colonoscopy is recommended from the age of 15 years and repeated every 3 years if no polyps are found or every year if colonic polyps are found. Other HHT-patients (non-SMAD4) should be screened for colon cancer as general population [6].

The different arteriovenous malformations associated with HHT are summarized in Table 1.

**Table 1:** Summary of different visceral arteriovenous malformations prevalence associated with HHT

AVM site	%
Hepatic	70%
Pulmonary	50%
Gastrointestinal	20%
Cerebral	10%
Skin teleangectasis	90%

There is no data in current literature about a comparative analysis of phenotypic manifestations among patients belonging to the same family unit – i.e. sharing a common genotype.

Therefore, the aim of our study is to determine, by describing the genotype-phenotype correlation in children with HHT and in their parents, whether HHT phenotypes are comparable among parents and affected children.

## Methods

We conducted a single-centre retrospective study on pediatric patients and their parents with a confirmed diagnosis of HHT. Data were collected between May 1_st_ 2022, and May 31_st_ 2023, from the medical records of Fondazione Policlinico Universitario A. Gemelli IRCCS (Rome, Italy).

Affected parents of pediatric patients were asked to respond to a standardized semi-structured questionnaire, facilitating further investigation into the phenotypic manifestations of the pathology.

Moreover, we conducted a standardized evaluation of all patients (including physical examination, medical history, laboratory and imaging tests performed) considering the following data:

- Clinical phenotype, investigated through the evaluation of the presence or absence of epistaxis, telangiectasias, AVMs (pulmonary, hepatic, cerebral, gastrointestinal), and major bleeding (one of the following criteria: past treatment for bleeding and/or iron deficiency anemia – iron infusion/ blood transfusion);
- Hemoglobin, serum iron and ferritin values, analyzed for all pediatric and adult patients. Hemoglobin and ferritin levels differ between males and females during adolescence and adulthood [11, 12];
- Diagnostic tests -if performed-, such as ENT (ear-nose-throat) target examination, ultrasonography of the abdomen, MRI of the abdomen, brain MRI, chest CT scan, echocardiography with bubble test;
- Therapeutic interventions, such as iron infusions, laser ablation/cautery for epistaxis, embolizations, blood transfusions.

For “subjects with a defined diagnosis of HHT,” we mean those who had confirmation of the pathology by genetic testing [13].

Data were analyzed using GraphPad PRISM 9. Comparable variables were analyzed using Student’s t test/one way ANOVA. A 0,05 level of significance was considered for all statistical tests.

### Editorial policies and ethical considerations

This study was conducted in accordance with the Declaration of Helsinki and approved by the Ethics Committee of the Fondazione Policlinico Universitario A. Gemelli IRCCS (protocol number 6241/20, ID 2999, date of approval 20 February 2020). All patients and/or their parents/legal guardians signed informed consent forms.

This research received no specific grant from any funding agency.

### Demographic and clinical characteristics

**Fig. 1:**
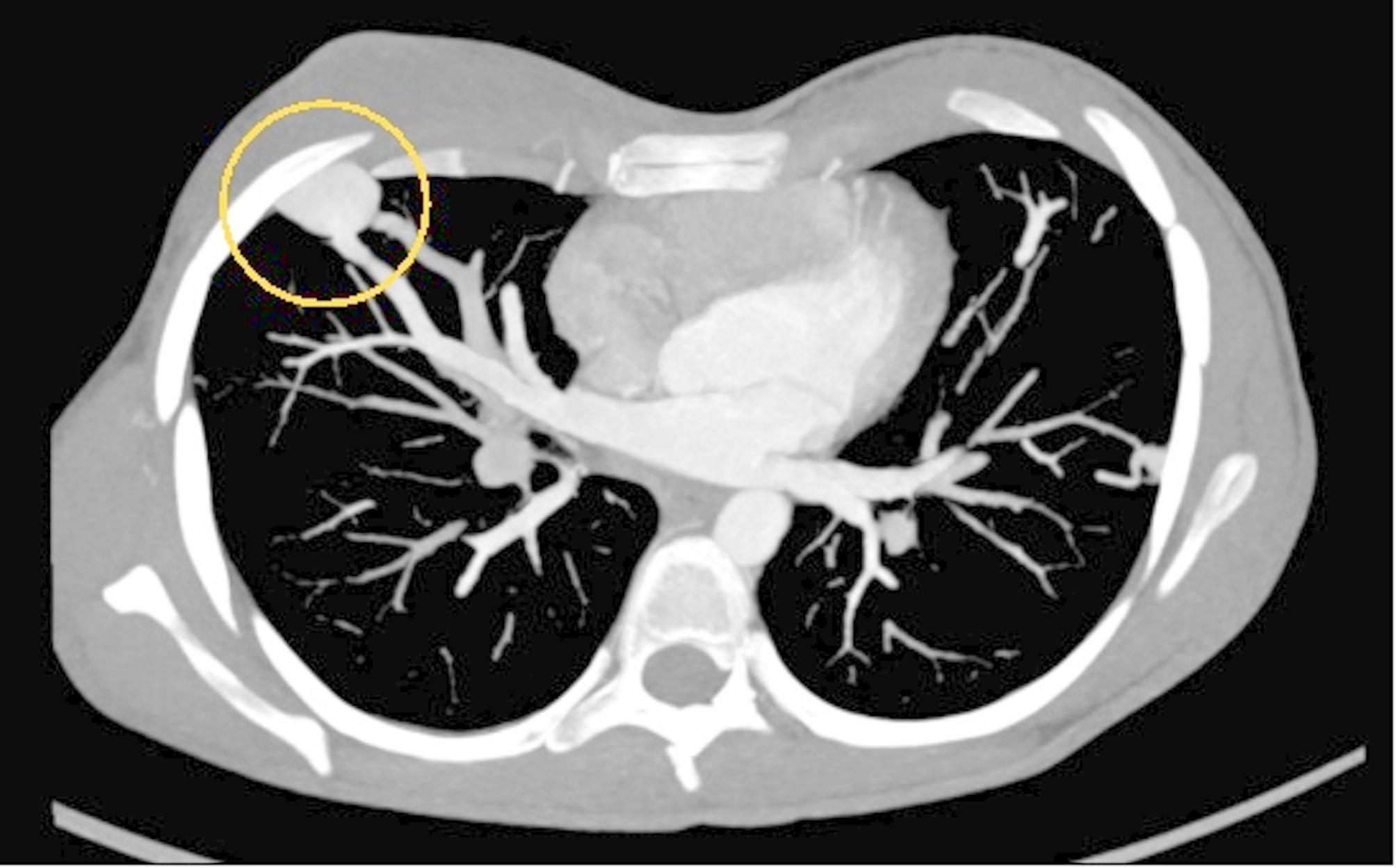
16-year-old child with a simple pulmonary fistula in the right lung characterized by a single 4 mm feeding artery (dot) and a large venous aneurysm (circle) without thrombotic apposition

**Fig. 2:**
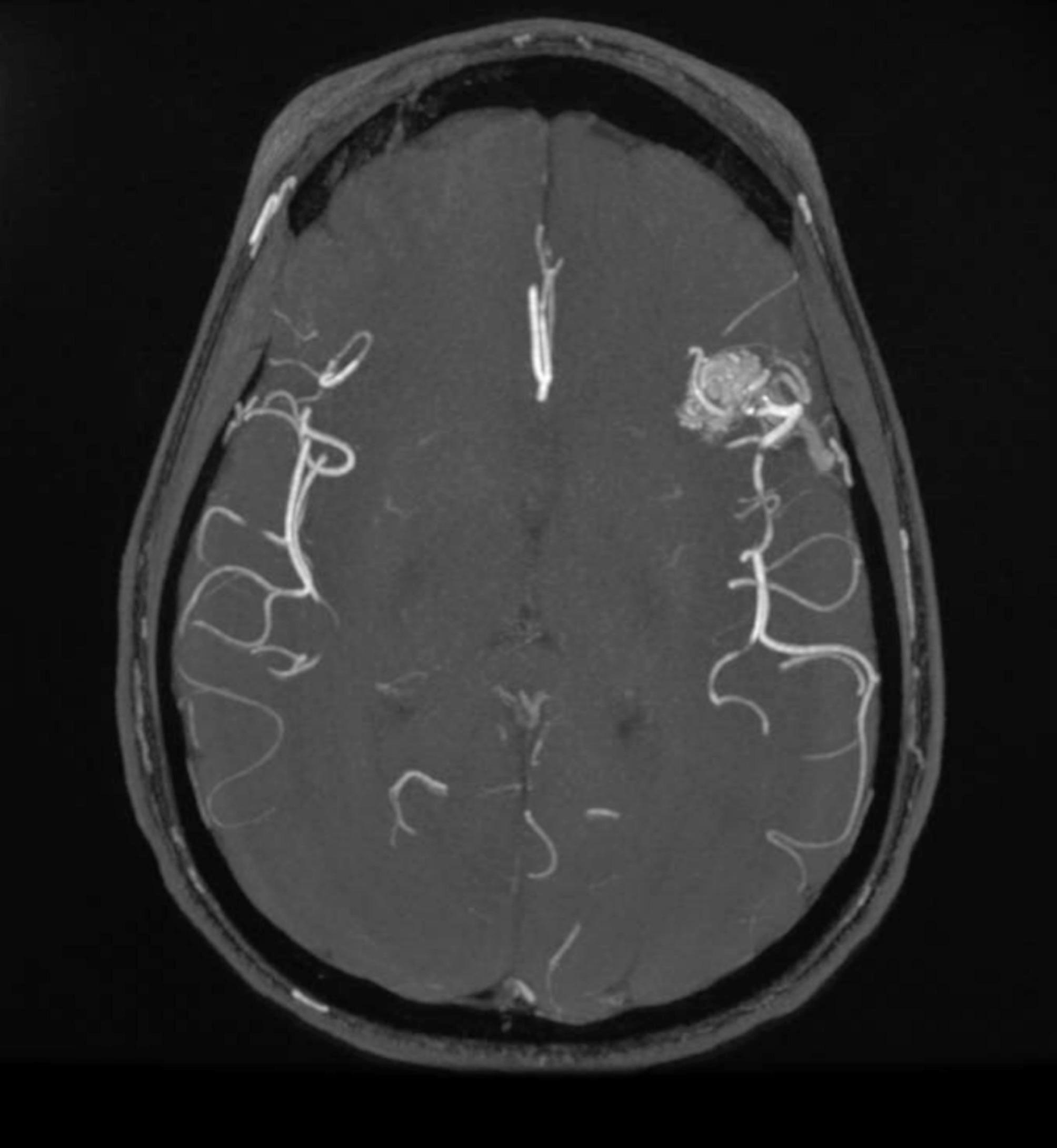
15-year-old child with a frontal lobe malformation with a 3 cm *nidus*

**Fig. 3:**
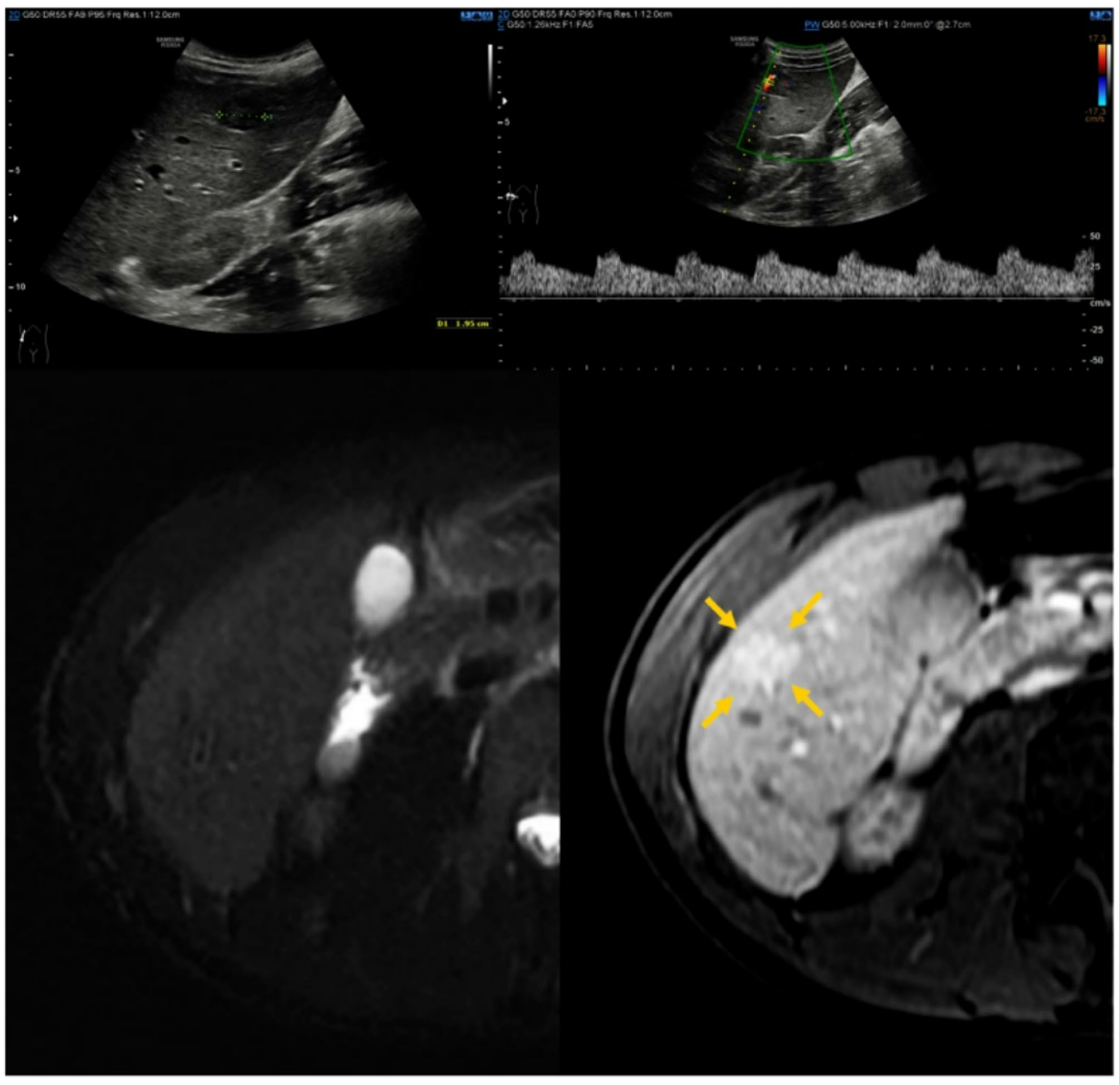
16-year-old child with a 20 mm liver vascular malformation between V and VI segment. US imaging shows the hypoechoic aspect of the lesion and the typical reduction of vascular resistances within the lesion. MR confirmed the vascular nature of the lesion

The pediatric population [Table 2] consists of 11 children aged between 0 and 20 years, including 6 boys and 5 girls, with an average age of 11.82 ± 5.51 years, as shown. Two couples of siblings are included (child 1 A-1B and child 4 A-4B). All patients (100%) have a positive family history of HHT. The clinical history of 10 patients (90.9%) – 5 males and 5 females – is characterized by the presence of epistaxis. In 4 patients (36.4%) – 2 males and 2 females – mucocutaneous telangiectasias were found, while AVMs are present in 3 patients (27.3%) – 2 males and 1 female. In particular, pulmonary AVM were diagnosed in 3 patients (27.3%), hepatic AVM in 1 (9.1%), cerebral AVM in 2 (18.2%) [Figs. 1, 2 and 3].

**Table 2:** Demographic and clinical characteristics of the population; statistically significant differences are marked with *

	Children	Adults
Mean age (years ± SD)	11,82 ± 5,51	47 ± 8,68
Sex (male/female ratio)	6/5	6/3
Epistaxis (N; %)	10/11; 90,9%	9/9; 100%
Mucocutaneous telangiectasias (N; %)	4/11; 36,4%	9/9; * 100%
Family history of HHT (N; %)	11/11; 100%	8/9; 88,9%
AVM (N; %)	3/11; 27,3%	5/9; 55,6%
Pulmonary AVM (N; %)	3/11; 27,3%	5/9; * 55,6%
Hepatic AVM (N; %)	1/11; 9,1%	2/9; * 22,2%
Cerebral AVM (N; %)	2/11; 18,2%	2/9; 22,2%

The adult population [Table 2] – i.e. affected parents who share the same genetic variant as pediatric patients – consists of 9 patients, including six males and three females, with an average age of 47 ± 8.68 years. Of these, 8 patients (88.9%) have a positive family history of HHT. In all patients (100%), epistaxis was found. 9 (100%) patients present mucocutaneous telangiectasias, while AVM are described in 5 patients (55.6%). In particular, pulmonary AVM were diagnosed in 5 patients (55.6%), hepatic AVM in 2 (22.2%), cerebral AVM in 2 (22.2%).

The diagnostic tests conducted in both populations are as follows:

- ENT the physical examination (the exam of nose, lips, oral cavity for telangiectases), performed in 10 out of 11 (90.9%) children and 8 out of 9 (88.9%) adults;
- Ultrasonography of the abdomen, conducted in 10 out of 11 (90.9%) children and in all 9 (100%) adults;
- MRI of the abdomen, conducted in 1 out of 11 (9.1%) children and in 1 out of 9 (11.1%) adults;
- Brain MRI, performed in 8 out of 11 (72.7%) children and 5 out of 9 (55.6%) adults;
- Chest CT scan, performed in 3 out of 11 (27.3%) children and 5 out of 9 (55.6%) adults, when screening tests were positive [6];
- Echocardiography with bubble test, performed in 4 out of 11 (36.4%) children and in 3 out of 9 (33.3%) adults.

### Genetic variants

All patients, children, and adults, have a genetic diagnosis. In 11 children, 3 (27.3%) have a *ENG* (NM_001114753.3) gene variant (OMIM #187300), and 8 (72.7%) have *ACVRL1* (NM_000020.3) variants (OMIM #600376). In 9 adults, 3 (33.3%) have an *ENG* variant, and 6 (66.7%) have *ACVRL1* variants.

## Results

### Laboratory values

To assess the impact of bleeding on anemia and iron reserves, the following laboratory values were considered: hemoglobin, serum iron and ferritin.

Within the pediatric population, no patient (0%) reported hemoglobin values below the cutoff [11]. In the adult population, 2 patients (22.2%) reported hemoglobin values below the cutoff [12].

No pediatric (0%) patient reported lower serum iron values than the cutoff [11]. 2 adult patients (22%) reported lower serum iron values than the cutoff [12].

No pediatric patient (0%) reported ferritin values below the cutoff [12]. 3 (33.3%) adult patients reported ferritin values below the cutoff [12].

### Therapeutic interventions

Considering the therapeutic interventions (iron infusions, laser ablation/cautery for epistaxis, embolizations, blood transfusions) to which the pediatric population has been subjected, it appears that no child (0%) has ever needed transfusions for reasons related to HHT, while 2/11 children (18.2%) have had their AVM embolized.

Considering the therapeutic interventions to which the adult population has been subjected, it appears that 1/9 adult (11.1%) has resorted to transfusions and 4/9 (44.4%) to iron infusions due to HHT-related anemia. 3/9 adults (33.3%) have also had their AVM embolized.

### Major bleeding

A comparison between the number of children and adults who have experienced major bleeding in the oral and nasal cavity shows that no child (0%) has experienced such bleeding, and 4 adults (44.4%) have required Emergency Room access for this reason.

### Genotype-phenotype correlation

Table 3 illustrates genotype-phenotype correlations among pediatric patients and their affected parents, considering the following clinical data for evaluating phenotypic expression:

**Table 3:** Genotype-phenotype correlation: child vs. adult

Gene	Variant	Zygosity		Classification ^****^				AVMs (1/0)	Treatments (1/0)		Major bleedings (1/0)	
*ACVRL1*	c.1435 C > T (p.Arg479Ter)		Het	Pathogenic	Family 1		Child 1 A	0		0		0
							Child 1B	0		0		0
							Parent 1	0		0		0
	c.200G > A (p.Arg67Gln)		Het	Pathogenic/Likely pathogenic	Family 2		Child 2	0		0		0
							Parent 2	0		1 (I)		1
	c.526–6 C > G		Het	Variant of uncertain significance	Family 3		Child 3	0		0		0
							Parent 3	1 (P-H-GI)		1 (I)		1
	c.230G > A p.(Cys77Trp)		Het	Likely pathogenic	Family 4		Child 4 A	1 (P-H-C)		0**		0
							Child 4B	1 (P)		0***		0
							Parent 4	0		0		0
	c.230G > A p.(Cys77Trp)		Het	Likely pathogenic	Family 5		Child 5	0		0		0
							Parent 5	1 (H)		0		0
	c.1120 C > T p.(Arg374Trp)		Het	Pathogenic/ Likely pathogenic	Family 6		Child 6	0		0		0
							Parent 6	0		0		0
ENG	c.893del p.(Gly298Alafs*61)		Het	Pathogenic	Family 7		Child 7	0		0		0
							Parent 7	1 (P)		1 (T, I, E)		1
	c.298del p.(Ser100Valfs*2)		Het	Pathogenic	Family 8		Child 8	0		0		0
							Parent 8	1 (P-H-C)		1 (E)		0
	c.1 A > G (p.Met1Val)		Het	Pathogenic	Family 9		Child 9	1 (P-C)		0		0
							Parent 9	1 (P-C)		1 (E)		1

^†^Legend:

• AVM: *P* = pulmonary; H = hepatic; C = cerebral; GI = gastrointestinal;

• Treatments: T = transfusions due to HHT-related anemia; I = iron infusions due to HHT-related anemia; E = embolization

• Heterozygous: Het

**: 1 iron infusion due to favism comorbidity

***: 1 blood trasfusion due to favism comorbidity

****: doi: 10.1038/gim.2015.30

- Presence of AVM (pulmonary, hepatic, cerebral, gastrointestinal);
- Treatments necessitated by the severity of anemia and/or AVM (iron infusions, laser ablation/cautery for epistaxis, embolizations, blood transfusions);
- Major bleeding (one of the following criteria: past treatment for bleeding and/or iron deficiency anemia – iron infusion/ blood transfusion).

It follows that:

- In family 1, with c.1435 C > T variant in *ACVRL1*, no AVM, major treatments, or bleeding were recorded, neither in the parent nor in the two children.
- In family 2, with c.200G > A variant in *ACVRL1*, no AVM, major treatment or bleeding was recorded in the child, while iron infusions and major bleedings in the parent were recorded.
- In family 3, with c.526–6 C > G variant in *ACVRL1*, no AVM, major treatments or bleeding was recorded in the child, while pulmonary, hepatic, and gastrointestinal AVMs, iron infusions, and major bleedings in the parent were recorded.
- In family 4, with c.230G > A variant in *ACVRL1*, no AVM, major treatment or bleeding was recorded in the parent, while pulmonary, hepatic, and cerebral AVMs were recorded in one child and pulmonary AVMs in the other.
- In family 5, with c.230G > A variant in *ACVRL1*, no AVM, major treatment or bleeding was recorded in the child, while hepatic AVMs were recorded in the parent.
- In family 6, with c.1120 C > T variant in *ACVRL1*, no AVM, major treatment or bleeding was recorded, neither in the parent nor in the child.
- In family 7, with c.893del variant in *ENG*, no AVM, major treatment or bleeding was recorded in the child, while pulmonary AVMs, blood transfusions, iron infusions, embolizations and major bleedings in the parent were recorded.
- In family 8, with c.298del variant in *ENG*, no AVM, major treatment or bleeding was recorded in the child, while pulmonary, hepatic, and cerebral AVMs and embolizations in the parent were recorded.
- In family 9, with c.1 A > G variant in *ENG*, pulmonary and cerebral AVMs were recorded in the child and pulmonary and cerebral AVMs, embolizations, and major bleedings were recorded in the parent.

Also, Table 3 illustrates the distribution of pulmonary, hepatic and cerebral AVM in the pediatric and adult population in relation to the genotype (*ACVRL1* or *ENG* variants).

Out of a total of 8 children and 6 adults with *ACVRL1* variants, 2 pediatric patients (25%) and 1 adult patient (16.7%) have pulmonary AVMs.

Out of a total of 3 children and 3 adults with *ENG* variants, 1 pediatric patient (33.3%) and 3 adult patients (100%) have pulmonary AVMs.

In 1 pediatric patient (12.5%) and no adult patient (0%) with *ACVRL1* variants, cerebral AVM were found. Among patients with *ENG* variants, however, 1 child (33.3%) and 2 adults (66.7%) have cerebral AVMs.

## Discussion

### Comparison with literature

The analysis of the phenotypes of the pediatric population examined revealed a series of evidence confirming the literature. 90.9% of patients have epistaxis, that is confirmed to be the first symptom to manifest itself during childhood, which, however, rarely requires cauterization or other significant interventions to control bleeding [9], as demonstrated by the absence of major bleedings (0%) in the target population.

36.4% of pediatric patients have mucocutaneous telangiectasias, which, when compared to the prevalence of those in the adult population (100%), has their later presentation confirmed [9]. The same applies to AVMs, present in 27.3% of pediatric patients and 55.6% of adult patients.

The correlation of the phenotype characterized by the presence of pulmonary and cerebral AVMs with genotypes characterized by *ENG* and *ACVRL1* variants is reported in Table 3. The increased frequency of pulmonary and cerebral AVMs in patients - both pediatric and adult - with *ENG* variants has been confirmed, as reported in literature [8]. However, as evidenced by the presence of cerebral AVMs in 1 pediatric patient (12.5%) with a *ACVRL1* variant, it must be reiterated that the genotype alone cannot and must not guide the screening practices, as different AVMs are potentially present in all genotypes [8]. An important pediatric-specific consideration is the higher prevalence of cortical malformations in HHT children, with polymicrogyria described as the most common type [14–16].

### Analysis of laboratory values

Hemoglobin, serum iron, and ferritin below the respective cutoffs – as referred for age specific pediatric cut-off [11] - were not recorded for any pediatric patient. Moreover, none of these patients needed iron infusions or blood transfusions. These data strengthen published recommendations about blood test timeline both in adults and children with HHT [17]. An apparently normal value of hemoglobin in patients with pulmonary phenotype, as known, may depend on compensatory polycythemia. These patients, therefore, should be focused during the pubertal development period, due to the increase of iron requirement or increased loss – in post-menarche females.

### Genotype-phenotype correlation

There is a well-known correlation between genotype and phenotype in HHT. ENG mutations cause severe pulmonary AVMs and a higher incidence of gastrointestinal bleeding, *ACVRL1* mutations correlate to milder phenotype with fewer pulmonary complications but significant epistaxis and gastrointestinal issues, while SMAD4 mutations are associated with additional complications, including gastrointestinal polyps and an increased risk of colorectal cancer.

Comparing children’s phenotypes to their parents sharing the same genotype (Table 3), despite the intrinsic limits linked to specific considerations relative to a dynamic pathology, we have seen that, out of a total of 9 families analyzed, a similar clinical phenotype was recorded in 3 families (33.3%).

In this regard, the case of family 4 deserves to be reported. The history of this family began in 2018 with the suspicion of HHT in the eldest son following the finding of hypoxemia (SpO2 85%) and multiple pulmonary AVMs, 2 of which were treated with embolization. Further diagnostic investigations were simultaneously carried out in the father and in the second child, and both were found to be affected by HHT with c.230G > A variant in *ACVRL1* (shared by all three family members). It is interesting to note that, with the same clinical score (3) assigned through the Curaçao criteria, we were faced with extremely different clinical pictures. The firstborn (20 years) presented epistaxis and the so called “lung phenotype”. The second son (16 years old) also presented epistaxis, but more severe, and, in addition to pulmonary AVMs, a hepatic AVM and a cerebral AVM – i.e. a mixed phenotype. On the other hand, the father (58 years) presented only epistaxis - less severe than in the children - and meager telangiectasias. Therefore, the difference in the phenotypic expression of the disease is evident, as, while it has no impact, if not small, on the quality of life of the father, it has a great impact on that of both children. The story of this family can help reminding that intra-familial phenotypic variation is well-established; in fact, most disease-causing pathogenetic variants in *ACVRL1*, *ENG*, and *SMAD4* are null alleles that result in haploinsufficiency [18]. Clinicians dealing with families with HHT should keep this peculiarity in mind and eventually discuss it with the families. Clinical variability is expected in the setting of autosomal dominant diseases with variable expressivity, such as HHT. Nonetheless, genotype-phenotype correlations have been well described in adults with HHT [19], but much less in children [20, 21]. In addition, a direct comparison between the phenotypes of parents and children carrying the same gene mutation within the same family has never been performed. This is the added value of our study.

It is important to note that, in Italy, there is a National Health Care System providing a free screening and follow up program for rare diseases, including HHT. This program includes periodic abdominal ultrasound, that can be performed before the symptoms and/or signs of complications might appear.

Our study has some limitations that need to be addressed. This is a single center study including few patients, with observational aims. It follows that, when possible, statistical analysis has been performed but limited by the sample size. A further limitation of our study is the shorter observation period in children compared to their parents. However, it is important to underline that, in recent years, the therapeutic approach for these patients has evolved towards a more conservative strategy, which may partially explain the lower number of treatments observed in children.

Further studies are needed to better investigate these findings.

## Conclusions

Based on our study, it is not possible to talk about a phenotype overlap between HHT in children and their parents with the same genotype, confirming that HHT is a hereditary disease with variable expressiveness. Current guidelines are useful and valuable for children and, since complications themselves may represent the onset manifestation in pediatric patients, an early diagnosis is indispensable for their prevention. Genetic testing, early screening, and close follow-up are crucial for optimizing outcomes in children with HHT.

## Data Availability

The data that support the findings of this study are available from the corresponding author upon reasonable request.
